# Association between substantial unpaid caregiving and psychological well-being among college students in Florida

**DOI:** 10.1371/journal.pmen.0000641

**Published:** 2026-07-31

**Authors:** Joanna F. Mackie, Kevin Zou, Mary Schmidt-Owens, Julia N. Soulakova

**Affiliations:** 1 Department of Population Health Sciences, College of Medicine, University of Central Florida, Orlando, Florida, United States of America; 2 Student Health Services, University of Central Florida, Orlando, Florida, United States of America; National University of Singapore, SINGAPORE

## Abstract

Caregiving is a common responsibility in the United States, and about 1 in 5 Americans provide unpaid care for older adults or family members with disabilities. Many adults also provide care for their children. Despite substantial evidence about the potential impact of caregiving on caregivers’ mental health, most studies focus on middle-aged adults. Less is known about how caregiving affects the psychological well‑being of young adults, particularly college students. This study examined whether spending substantial time on unpaid caregiving (11 or more hours per week) is associated with greater psychological well‑being among students. The study participants were selected using a probability sample and included students aged 18–30 years who were enrolled at a large public university in Florida. A total of 692 participants completed the 2025 Spring American College Health Association–National College Health Assessment. Overall psychological well‑being was assessed using Diener’s Flourishing Scale. Statistical methods included Kruskal-Wallis tests and a generalized linear model with a Gamma distribution, controlling for demographic and academic characteristics. Overall, 3.61% of students reported spending substantial time on caregiving. The overall psychological well-being score (mean and median) was significantly higher among students who spent substantial time on caregiving compared to those who did not (p = 0.0177). The model yielded a similar conclusion (mean difference = 3.82, 95%CI = 1.15:6.49, p = 0.0135) and resulted in additional significant differences in mean scores across several characteristics, including sexual minority status (p < 0.0001) and race/ethnicity (p = 0.0020). Findings suggest that spending substantial time on caregiving is associated with greater psychological well-being among college students. Further research is needed to identify the mechanisms underlying this association and to examine whether these findings generalize to other populations.

## 1. Introduction

Caregiving activities can include unpaid care provided to children or other family members, as well as unpaid care provided to friends, neighbors, or other individuals with health-related or functional needs [1,2]. Family caregiving typically refers to the unpaid, informal care provided by relatives or close friends to individuals who require assistance with daily functioning due to chronic illness, disability, or aging [1,2]. In the United States, about 63 million Americans were family caregivers in 2025 [2]. Family caregivers provide personal care, household management, and complex medical tasks traditionally performed by trained professionals [1,2]. In addition to family caregiving, caring for dependent children represents another major form of caregiving responsibility among adults. Notably, this responsibility extends to the undergraduate student population, where roughly 20% of individuals had at least one child under age 18 in 2020 [3]. Caregivers play a central role in supporting young children’s development, helping them acquire foundational skills such as motor function, communication, emotional regulation, and social interactions [4]. Although older children may require less intensive care, caregivers continue to provide essential support for schoolwork, resilience, and healthy behaviors [5]. In this study, caregiving is defined broadly to include care for children or other family members.

While caregiving could be described by caregivers as rewarding [6], caregiving responsibilities can also disrupt goal setting and personal priorities, and affect mental health. For example, caregiving was significantly associated with frequent mental distress and life dissatisfaction among U.S. adults [7]. Likewise, among young adults, caregiving may lead to increased emotional and physical stress and social isolation, which can negatively affect mental health, and, in turn, influence decisions about education and career pathways [8–10]. For example, a study of 18–24 year-old undergraduate students found that depression and anxiety levels were higher among participants who had caregiving responsibilities before college (regardless of whether those responsibilities continued during college), compared with peers who had never had caregiving responsibilities [11].

In summary, the literature suggests that providing caregiving during young adulthood, including the college years, does not have uniformly positive or negative effects on caregivers’ psychological well-being [10]; rather, its impact likely depends on individual and contextual factors. This highlights the need for more research on college students, who remain underrepresented in caregiving studies despite the potential for caregiving experiences to shape their developmental trajectories and psychological well-being [12,13].

Our primary goal was to evaluate whether spending substantial time on unpaid caregiving (11 hours or more per week) is associated with higher levels of psychological well-being among college students in Florida, and whether this association remains significant after adjusting for students’ demographic and academic characteristics. This investigation was motivated by prior studies demonstrating disparities in mental health by demographic and academic factors among adults [14–18]. Our secondary goal was to identify the demographic and academic characteristics that were significantly associated with greater levels of the overall psychological well-being.

## 2. Materials and methods

### 2.1. Data and measures

To assess the study goals, we used data from the 2025 Spring American College Health Association (ACHA)—National College Health Assessment (NCHA). The study participants were selected using a probability sample and included students aged 18–30 years who were enrolled at a large public university in Florida. The ACHA administered the assessment online in March of 2025. The survey was open for three weeks and was completed anonymously. Five reminders were emailed to non-respondents within this period. All respondents were given the opportunity to participate in a drawing, and 15 respondents were awarded a $100 gift card. The assessment was completed by 726 students and the response rate, as computed by the ACHA, was 7.3%. The ACHA prepared the de-identified dataset. The authors had no access to information that could be used to identify the study participants during or after the data collection. The authors accessed the data on April 24, 2025. The analytic sample included 692 students who reported complete information on all study measures. The study was determined to be exempt by the Institutional Review Board.

The primary measure was overall psychological well-being (or flourishing), assessed using Diener’s scale [19]. The scale consists of eight items, each self-rated on a 7-point scale (1 = strongly disagree to 7 = strongly agree): (1) I lead a purposeful and meaningful life, (2) My social relationships are supportive and rewarding, (3) I am engaged and interested in my daily activities, (4) I actively contribute to the happiness and well-being of others, (5) I am competent and capable in the activities that are important to me, (6) I am a good person and live a good life, (7) I am optimistic about my future, and (8) People respect me. The overall psychological well-being score ranges from 8 to 56, with higher score indicating greater well-being. The overall score and eight individual items were treated as dependent variables in all analyses.

The primary independent variable, substantial unpaid caregiving (0–10 hours per week versus 11 + hours per week), was defined using responses to the survey item “How many hours do you spend in a typical week (7 days) on the following activities? Taking care of children or other family members (unpaid).” The response options included the following categories: “0 hours,” “1-5 hours,” “6-10 hours,” “11-15 hours,” “16-20 hours,” “21-25 hours,” “26-30 hours,” and “More than 30 hours.” The cutoff point of 11 hours was selected to capture a substantively meaningful level of caregiving time commitment while preserving adequate sample size of the substantial caregiving group. We note that substantial upaid caregiving is defined in a broad sense and includes different types of caregiving activities. Additional independent variables included the demographic and academic characteristics listed in Table 1.

**Table 1:** Sample Description (n = 692); 2025 Spring American College Health Association—National College Health Assessment Data Collected at a Public University in Florida.

Characteristic	Group	Group Sample Size	%
**Demographic characteristics**			
Age^a^	18-20 years old	317	45.81
	21-24 years old	290	41.91
	25-30 years old	85	12.28
Sex	Female	474	68.50
	Male	218	31.50
Sexual minority status	Sexual minority	208	30.06
	Straight	484	69.94
Race/ethnicity^c^	Non-Hispanic White	288	41.62
	Non-Hispanic Other	213	30.78
	Hispanic	191	27.60
Relationship status	Not in a relationship	395	57.08
	In a relationship: married/partnered or in relationship not married/partnered	297	42.92
**Academic characteristics**			
Program type	Undergraduate	597	86.27
	Graduate	95	13.73
Overall Grade Point Average^d^	A	411	59.39
	B, C, or below	281	40.61
**Substantial Unpaid Caregiving**			
Substantial unpaid caregiving	0-10 hours per week	667	96.39
	11 + hours per week	25	3.61

^a^Only one student reported being 30 years old.

^b^The sexual minority group included bisexual (n = 98), gay/lesbian (n = 30) and other sexual minority (n = 80) students.

^c^The Non-Hispanic Other group included students who were non-Hispanic and identified as Black/African American (n = 71), Asian/Asian American (n = 84), biracial or multiracial (n = 26), or other (n = 32).

^d^The “B, C, or below” group included students with an overall Grade Point Average of B (n = 246) and those with C or below (n = 35).

### 2.2. Statistical analysis

First, we examined the distributions of the dependent variables. Because the normality assumption was questioned, we used nonparametric tests to assess the significance of associations between unpaid caregiving and each dependent variable. Specifically, Kruskal-Wallis tests were conducted at a 5% significance level [20]. Effect sizes were computed using epsilon-squared, which ranges from 0 (no relationship) to 1 (perfect relationship) [21]. The distribution of the overall psychological well-being score was left-skewed. While simpler models and alternative data transformations were initially considered, the final model was a generalized linear model with a Gamma distribution and log link fitted to the reflected score, defined as 64 minus the original overall psychological well-being score. The application of such models in psychology has been previously addressed [22]. The model controlled for demographic characteristics, including students’ age, sex, race/ethnicity, sexual minority status, and relationship status, as well as for academic factors such as GPA and program type (undergraduate, graduate). The overall model fit was assessed using goodness-of-fit criteria, e.g., deviance divided by degrees of freedom (df) substantially different from 1.0 may indicate potential model misspecification, and a deviance residual plot. In addition, influential observations were assessed using leverage and Cook’s distance; observations were flagged for review if their leverage values or Cook’s distance values exceeded 0.05. Significance of each predictor was assessed at a 5% significance level. The model was used to compute 95% confidence intervals (CIs) for the differences between the mean reflected scores. To obtain the corresponding CIs for the differences between the mean original scores, the confidence limits were multiplied by -1, which reversed the lower and upper bounds. Bonferroni adjustments for multiplicity were used for predictors with three or more levels. Statistical computing was performed using PROC GENMOD, the NLMEANS macro, and other procedures in SAS 9.4 software and SAS OnDemand for Academics [23].

## 3. Results

Table 1 presents the sample description in terms of demographic and academic characteristics, along with substantial unpaid caregiving. Most students (82.66%, n = 572) reported spending no time on caregiving, followed by 11.27% (n = 78) who reported spending 1–5 hours per week and 2.46% (n = 17) who reported 6–10 hours per week. Only 3.61% (n = 25) of students reported spending substantial time on caregiving (11 or more hours per week), see Table 1.

Table 2 provides summary statistics for all dependent variables by caregiving group, along with the results of Kruskal-Wallis tests. Higher mean values corresponded to students who spent substantial time on caregiving compared with those who did not. Statistically significant differences between groups were observed for the overall psychological well-being score and four of the eight individual items: “I actively contribute to the happiness and well-being of others,” “I am competent and capable in the activities that are important to me,” “I am a good person and live a good life,” and “people respect me.” Nonetheless, the corresponding effect sizes were very small (approximately 0.01), indicating limited practical significance.

**Table 2:** Results of Bivariate Analysis (n = 692); 2025 Spring American College Health Association—National College Health Assessment Data Collected at a Public University in Florida.

Dependent Variable (Kruskal-Wallis Test Result)^a^	Mean (Standard Error) and Median	
	Students who spend 0–10 hrs/wk on caregiving (n = 667)	Students who spend 11 + hrs/wk on caregiving (n = 25)
**Individual Items**		
I lead a purposeful and meaningful life (p = 0.2666)	5.51 (0.05), 6.00	5.72 (0.30), 6.00
My social relationships are supportive and rewarding (p = 0.2537)	5.69 (0.05), 6.00	6.00 (0.20), 6.00
I am engaged and interested in my daily activities (p = 0.0645)	5.33 (0.05), 6.00	5.76 (0.28), 6.00
**I actively contribute to the happiness and well-being of others (χ**^**2**^ **= 8.02, df = 1, p = 0.0046)**	**5.71 (0.04), 6.00**	**6.28 (0.21), 7.00**
**I am competent and capable in the activities that are important to me (χ**^**2**^ **= 5.84, df = 1, p = 0.0157)**	**5.82 (0.04), 6.00**	**6.32 (0.18), 7.00**
**I am a good person and live a good life (χ**^**2**^ **= 8.27, df = 1, p = 0.0040)**	**5.77 (0.05), 6.00**	**6.32 (0.24), 7.00**
I am optimistic about my future (p = 0.1382)	5.54 (0.06), 6.00	5.80 (0.33), 6.00
**People respect me (χ**^**2**^ **= 6.22, df = 1, p = 0.0126)**	**5.52 (0.05), 6.00**	**6.12 (0.21), 6.00**
**Overall Score**		
**Overall psychological well-being score (χ**^**2**^ **= 5.63, df = 1, p = 0.0177)**	**44.89 (0.32), 47.00**	**48.32 (1.44), 50.00**

^a^NS indicates statistically non-significant differences; values in bold indicate statistically significant differences.

The generalized linear model showed an adequate overall fit based on goodness-of-fit criteria (deviance/df = 0.17, Pearson chi-square/df = 0.17, scaled deviance/df = 1.04, and scaled Pearson chi-square/df = 1.05) and the deviance residual plot, which showed a random scatter of points around zero with no systematic patterns. Assessment of case-level diagnostics revealed no influential observations. Indeed, while a few observations exceeded the leverage threshold of 0.05 (max = 0.08), they did not substantially affect the estimates, as Cook’s distance values were uniformly low (max = 0.03). Table 3 presents the results based on the model. After adjusting for other factors, substantial unpaid caregiving was significantly associated with overall psychological well-being, with a higher mean score for students who spend 11 + hours per week on caregiving compared with those who spend 0–10 hours per week.

**Table 3:** Model-Based Estimates for the Overall Psychological Well-Being Score (n = 692); 2025 Spring American College Health Association—National College Health Assessment Data Collected at a Public University in Florida.

Independent Variable^a^	Comparison	Mean Difference and 95% Confidence Interval for the Mean Difference^b^
Sexual minority status (p < 0.0001)	**Sexual minority vs. straight**	**-3.95 (-5.29, -2.60)**
Race/ethnicity (p = 0.0020)	**Hispanic vs. Non-Hispanic White**	**1.50 (0.02, 2.98)** ^**b**^
	Non-Hispanic Other vs. Non-Hispanic White	-1.10 (-2.69, 0.50)^b^
Relationship status (p = 0.0101)	**In a relationship vs. not in a relationship**	**1.51 (0.36, 2.66)**
Overall Grade Point Average (p < 0.0001)	**A vs. B, C, or below**	**2.50 (1.29, 3.71)**
Substantial unpaid caregiving (p = 0.0135)	**11 + hours per week vs. 0–10 hours per week**	**3.82 (1.15, 6.49)**

^a^The model included additional covariates that were not statistically significant: age (p = 0.5338), sex (p = 0.0791), and program type (p = 0.2652).

^b^Values in bold indicate statistically significant differences. For race/ethnicity, a 95% simultaneous confidence band using the Bonferroni adjustment is reported.

Table 3 also shows that the mean overall psychological well-being score varied significantly by sexual minority status, race/ethnicity, relationship status, and Grade Point Average (GPA). However, there were no significant differences in the mean score by age, sex, and program type (undergraduate, graduate).

## 4. Discussion

The primary finding of our study was that among college students aged 18–30 years, providing substantial unpaid caregiving (11 + hours per week) was associated with higher levels of psychological well-being, even after adjusting for key demographic and academic characteristics. In addition, providing substantial unpaid caregiving was positively associated with four of the eight individual items, reflecting subjective facets of well-being such as prosocial contribution (“I actively contribute to the happiness and well-being of others”), competence (“I am competent and capable in the activities that are important to me”), personal integrity (“I am a good person and live a good life”), and social respect (“people respect me”). While these findings should be viewed as exploratory due to the item-level nature of the analysis and small effect sizes, they are consistent with prior literature suggesting that, in addition to negative aspects of caregiving during emerging adulthood [8–11], caregiving may also foster psychosocial strengths [10], including self-perceived social contribution and respect, competence, and personal integrity.

Recognizing both the challenges faced by caregiving students and the psychological strengths associated with caregiving could help universities develop support programs that address caregiving demands while promoting student well-being. To provide support for caregiving demands, university student services might ask students to report caregiving status upon enrollment and collect relevant information, such as the type of caregiving provided. Then tailored resources could be offered, such as additional support in navigating caregiving students’ academic goals, opportunities for peer connection among caregivers, and assistance in identifying caregiving resources in the local community [10].

The secondary findings indicated that overall psychological well-being varied across several explored demographic and academic characteristics, including sexual minority status, race/ethnicity, relationship status, and GPA. These differences are broadly consistent with prior literature. First, the greater well-being observed among students who identified as straight compared with those who identified as members of sexual minority communities aligns with research documenting higher levels of stress and related mental health challenges among sexual minority adults compared with heterosexual adults [14,16,24,25]. Second, although studies on mental health disparities by race/ethnicity have yielded mixed results, our finding that psychological well-being is greater among Hispanic students compared with non-Hispanic White students is consistent with research indicating lower lifetime prevalence of mental disorders among Hispanic adults compared with White adults [18]. Third, while the association between relationship status and mental health depends on the quality and context of the relationship, our finding that being in a relationship is associated with greater well-being aligns with prior research showing that being in a happy relationship is linked to higher subjective well‑being [26]. Finally, the greater well-being observed among students with higher GPA compared to those with lower GPA is consistent with prior studies demonstrating a positive association between academic functioning and mental health, even though prior research often examined the reverse link, e.g., between mental health symptoms and academic performance [27,28].

The study has several limitations related to its design and statistical methods. First, although we identified a significant association between spending substantial time on unpaid caregiving and psychological well-being, the cross-sectional nature of the survey prevents any conclusions regarding causality. In addition, the observed association may reflect pre-existing differences between students who do and do not spend substantial time on unpaid caregiving. Indeed, individuals with stronger time management skills, greater family connectedness, and a stronger sense of purpose may be more likely to assume substantial unpaid caregiving roles. Moreover, the findings are limited to the two caregiving time commitment groups considered (0–10 hours per week versus 11 + hours per week) and the specific comparisons made. Consequently, the observed associations may differ across subgroups; for example, the overall association between substantial unpaid caregiving and psychological well-being could vary by race/ethnicity [29,30]. Such subgroup analyses were not conducted due to the limited sample size of the substantial unpaid caregiving group. Second, all measures were self-reported, which may introduce recall or social desirability bias, particularly for sensitive information [31–33]. Third, the sample included only 18–30 year-old college students enrolled in a public university in Florida, which limits the generalizability of the findings to the college student population at the source university and to other populations or regions. A related limitation is the low response rate of 7.3%, despite efforts by the source university and ACHA to increase participation, such as sending several reminders. The low response rate may partially reflect the limited incentives offered to participants, Finally, although the generalized linear model with a Gamma distribution and log link function was appropriate for the distribution of the reflected psychological well-being score, the model was evaluated using specific diagnostic criteria. Using more conservative criteria could lead to different conclusions. In addition, the model results may be affected by reduced statistical power due to the small sample size of the substantial unpaid caregiving group and by confounding. For example, complexity of caregiving tasks, which may influence mental health outcomes [34], was not accounted for in this study.

This study highlights the need to expand research on young adult caregivers, a population that is often overlooked in U.S. caregiving policy and surveillance systems. First, future longitudinal studies are needed to determine the over-time relationship between substantial unpaid caregiving and psychological well-being. Second, comprehensive assessments are needed to identify the factors that shape caregivers’ experiences and perceptions of these experiences, such as the complexity and burden of caregiving tasks, financial well-being, cultural expectations, and family cohesion [34,35]. In addition, it is important to identify populations of young adult caregivers who may have limited access to mental health resources and support [34], with the primary goal of developing targeted interventions and policies that support their well-being and resilience. These efforts are critical within both university and broader community settings, where tailored support systems may be particularly beneficial for young adult caregivers.
